# Screening and prenatal diagnosis of survival motor neuron gene deletion in pregnant women in Zhaoqing city, Guangdong Province

**DOI:** 10.1186/s12920-023-01468-0

**Published:** 2023-03-01

**Authors:** Zhiwei Huang, Qingchan Yang, Jianxiang Ye, Jianxing Huang, Jin Lin, Jing Chen, Zizhao Liang, Zijie Cao

**Affiliations:** 1 Clinical Laboratory, The Second People’s Hospital of Zhaoqing, Zhaoqing, The People’s Republic of China; 2 Obstetrical Department, The Second People’s Hospital of Zhaoqing, Zhaoqing, The People’s Republic of China; 3 Prenatal Diagnosis Center, The Second People’s Hospital of Zhaoqing, Zhaoqing, The People’s Republic of China; 4 Clinical Laboratory, The Second People’s Hospital of Zhaoqing, Zhaoqing, The People’s Republic of China; 5 Clinical Laboratory, The Second People’s Hospital of Zhaoqing, No. 2, Jiansheer Street, 526000 Zhaoqing City, Guangdong province The People’s Republic of China

**Keywords:** Spinal muscular atrophy, *SMN1*, Carrier screening, Prenatal diagnosis

## Abstract

**Objective:**

A total of 5,200 pregnant women in Zhaoqing city, Guangdong Province, were screened to identify spinal muscular atrophy (SMA) mutation carriers to guide the prevention of SMA and prevent the birth of children with SMA.

**Methods:**

Exons 7 and 8 (E7 and E8) of the survival motor neuron (SMN) 1 gene were detected in women using real-time fluorescence quantitative polymerase chain reaction. *SMN1* and *SMN2* copy numbers in those who were initially identified as carriers were verified via targeted region capture and next-generation sequencing. When both partners were identified as carriers, prenatal diagnosis of the fetus was performed.

**Results:**

Among the screened women, 75 SMA carriers (71 cases had both E7 and E8 heterozygous deletions and 4 cases only had an E7 heterozygous deletion) were identified, with a carrier frequency of 1.44% (95% confidence interval: 1.31–1.65%). Three couples where both spouses were identified as SMA carriers, and their three fetuses were subjected to prenatal genetic analysis. Of the three, one had homozygous deletions of E7 and E8 and the other two had heterozygous deletions of E7 and E8. After a detailed prenatal consultation, the former couple decided to terminate the pregnancy.

**Conclusion:**

Through screening and prenatal diagnosis of pregnant women in Zhaoqing city, Guangdong Province, the incidence of SMA can be reduced, prevention of birth defects can be improved, incidence of birth defects can be effectively minimized.

## Introduction

Spinal muscular atrophy (SMA) is a type of muscle weakness and muscle atrophy caused by motor neuron degeneration in the anterior horn of the spinal cord [1]. The clinical manifestations of SMA in children vary greatly and can be classified into four types according to the age of onset, motor function acquired by the patient, and the disease progression rate.

SMA, an autosomal recessive disease, has an onset probability of approximately 1 in 5,000 to 1/10,000 [2], and the carrier rate, which varies country and region wise, is approximately 1 in 35 to 1 in 85 [3–6]. The survival motor neuron (SMN) gene on chromosome 5q13.2, identified in 1995, is considered the pathogenic gene of childhood SMA [7]. *SMN* has two highly homologous copies on one chromosome: *SMN1* on the telomeric side, which is a functional gene, and *SMN2* on the centromeric side. Studies have shown that 95% of all *SMN1* deletions are in exons 7 (E7) and/or 8 (E8)[8].

Because of severe symptoms, a high fatality rate, and a clear association with pathogenic genes, SMA carrier screening has long been the interest of clinicians worldwide. In 2008, the American Society of Medical Genetics recommended that SMA carrier screening tests should be offered to all couples [9]. In 2017, the American College of Obstetrics and Gynecology recommended [10] that all women who are considering pregnancy or are already pregnant should be screened for SMA mutations. In 2019, nusinersen, a medication used for treating SMA, was launched in mainland China, and expert consensus on the genetic diagnosis of SMA was also published [11, 12]. At present, SMA mutation screening in both parents and newborns is being routinely performed in some countries and regions, along with some regions of China. In the present study, the SMA carrier screening of 5,200 pregnant women in Zhaoqing city, Guangdong Province, was conducted using real-time quantitative polymerase chain reaction (PCR). The frequency of SMA carriers in this area was demonstrated for the first time. In addition, prenatal diagnosis of high-risk fetuses was performed to reduce the number of births of children with SMA.

## Materials and methods

### Study population

From December 2020 to April 2022, pregnant women with the normal phenotype who were admitted to the Second People’s Hospital of Zhaoqing were informed about SMA carrier screening. This study and its protocols were approved by the Institutional Review Board of The Second People’s Hospital of Zhaoqing (approval no.: [2020] 001), and all patients signed the informed consent document before inclusion in the study.

### Genomic DNA extraction

Whole blood (2 mL) was collected in vials containing ethylenediamine tetraacetate acid, and DNA was extracted using a Lab-Aid 820 DNA extraction kit (Xiamen Zeesan Biotech Co., Ltd). The extracted DNA was assessed for purity (absorbance ratio of 260/280 nm between 1.8 and 2.0) and concentration using an UV spectrophotometer, and the final DNA concentration was adjusted to between 10 and 20 ng/µL.

### Real-time fluorescence quantitative PCR

Real-time fluorescence quantitative PCR was performed for primary screening. The homozygous deletions of E7 and/or E8 in *SMN1* in the DNA samples were detected using Shanghai Medicore Technology Co., Ltd., kit. The kit uses the minor groove binder probe technology in real-time fluorescence quantitative PCR, with the human ribonuclease P protein subunit P40 gene as the internal standard gene. Relative quantitative detection of the gene copy number was also performed. Real-time PCR analysis was divided into separate experiments for the two exons. The procedure was performed according to the manufacturer’s instructions and the results were evaluated using the cycle threshold (Ct) method. In the E7 and E8 reaction, ΔΔCt ≤ − 0.55 indicated normal and − 0.45 < ΔΔCt ≤ 0.45 indicated a heterozygous deletion. In the E7 reaction, ΔΔCt > 0.8 indicated a homozygous deletion of E7, and in the E8 reaction, ΔΔCt > 1.5 indicated a homozygous deletion of E8.

### Targeted region capture and NGS

Multiplex PCR was used for targeted region capture. After two rounds of PCR amplification and product purification, amplicon library was obtained and quantified using Qubit 3.0 Fluorometer (Thermo Fisher Scientific) and the library length was determined using Agilent Bioanalyzer 2100 (Agilent Technologies Inc.). The diluted library was sequenced with sequencing primers on Illumina NextSeq 500/550 platform (Illumina), with a mean depth of coverage of 100×. Raw sequencing image data files were converted into raw FASTQ files through image analysis and base calling. Adapters and low-quality reads were filtered, valid sequence reads were mapped to the hg19 human genome reference (Genome Reference Consortium GRCh37) via the Burrows-Wheeler Aligner software, and variant calling was performed using the Genome Analysis Toolkit software (GATK). The ANNOVAR software was used for variant annotation. The copy number of *SMN1* and *SMN2* was calculated according to the normalized read number and read number ratio of distinguished bases located in exon 7.

### Statistical analyses

SPSS 22.0 was used for statistical analyses. Data are presented as percentages, and the χ² test was used for comparison between groups. *P* < 0.05 indicated statistical significance. Previous studies published in China in recent years were retrieved to compare the population-carrying rate of SMA among different regions.

## Results

**Fig. 1:**
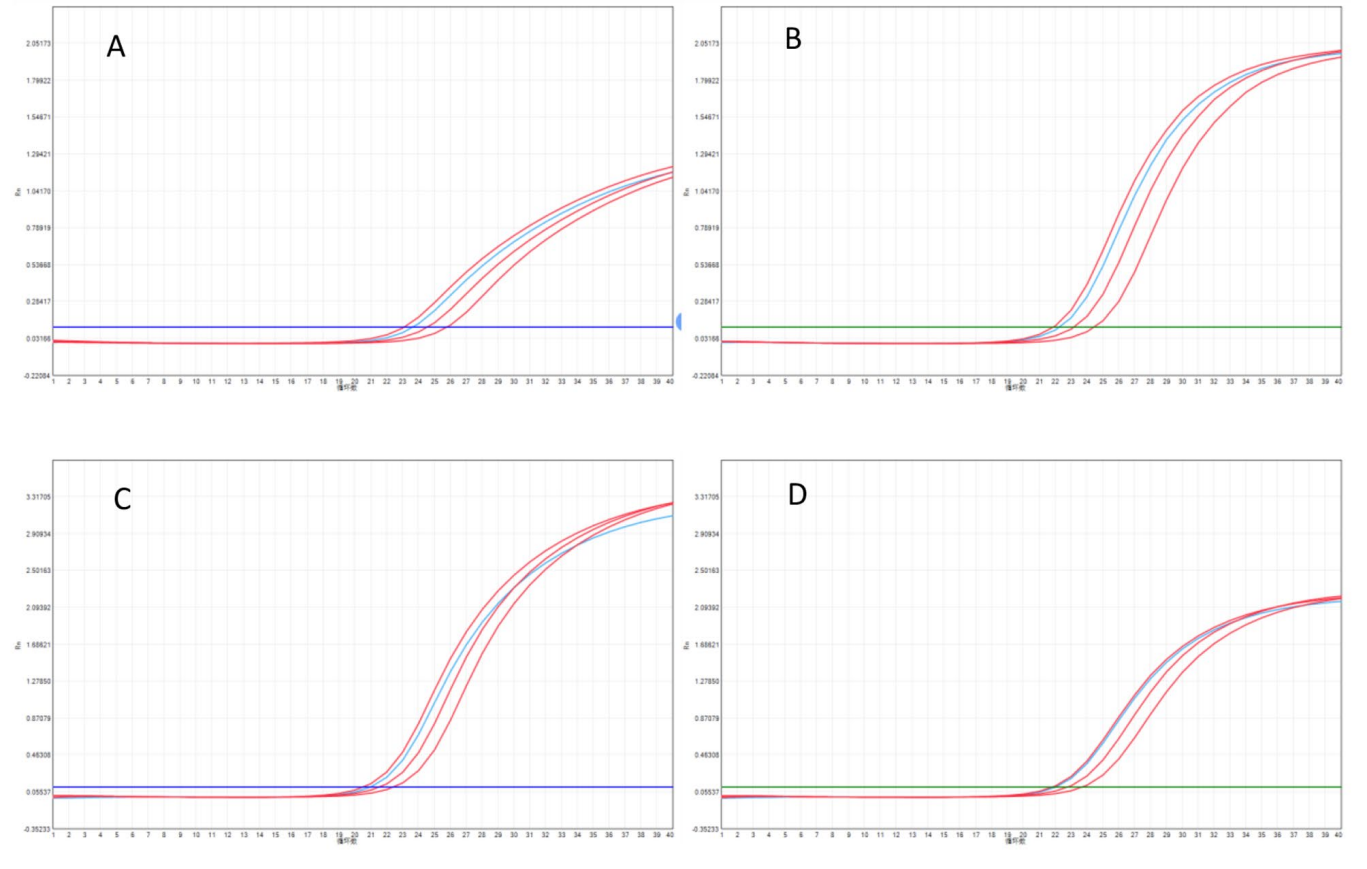
Real-time fluorescence quantitative PCR images of exon7 and 8 in pregnant women A: FAM channel for E7; B: VIC channel for E7; E7∆Ct_s = Ct_FAM-Ct_VIC = 23.61–22.23 = 1.38; E7∆Ct_a = 1.313; E7∆∆Ct=∆Ct_s-∆Ct_a = 0.0667, the result was an E7 heterozygous deletion; C: FAM channel for E8; D: VIC channel for E8; E8∆Ct_s = Ct_FAM-Ct_VIC = 20.96‒22.03=-1.07; E8∆Ct_a=‒1.293; E8∆∆Ct=∆Ct_s-∆Ct_a = 0.223, the result was an E8 heterozygous deletion

**Fig. 2:**
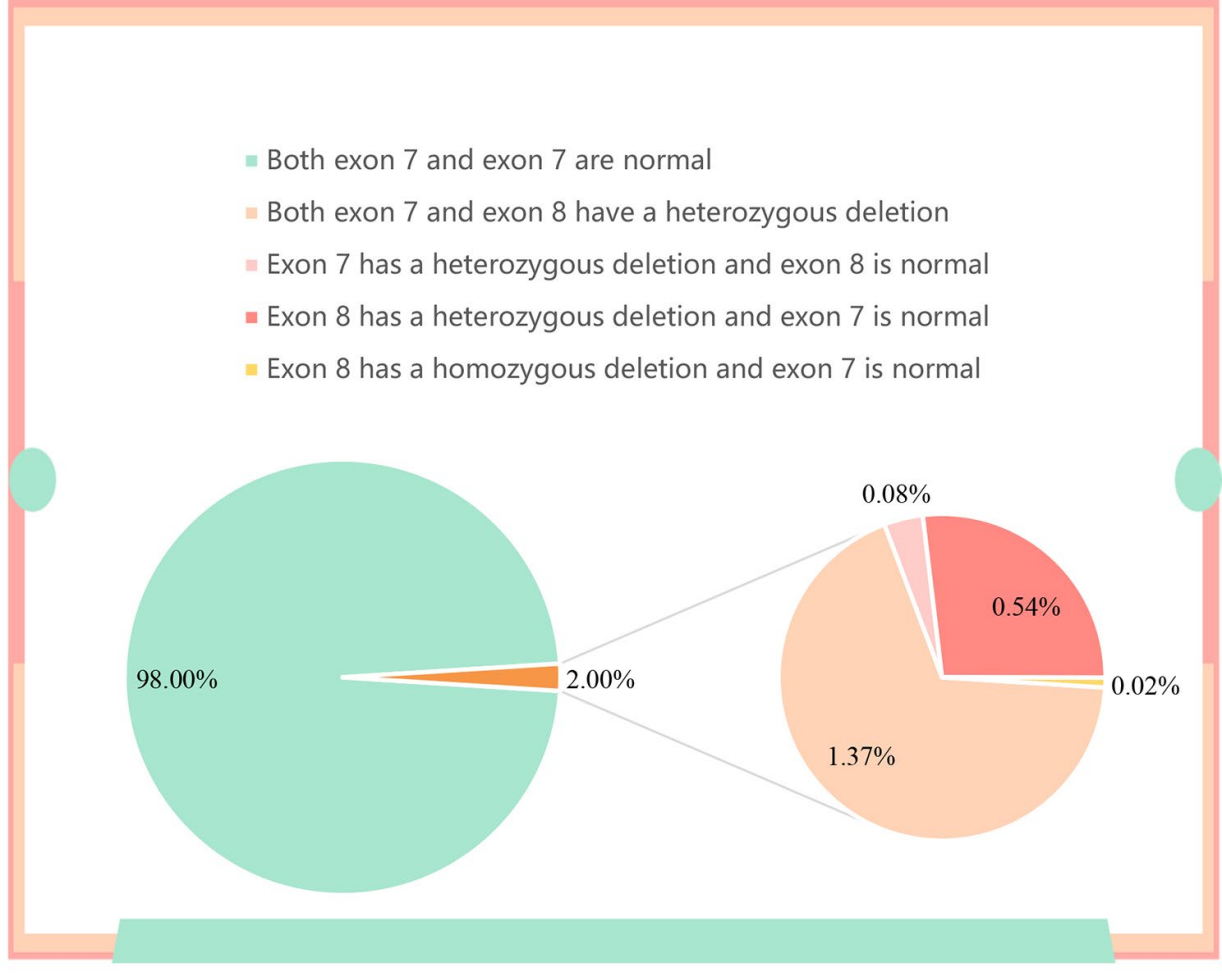
Genotyping analysis of spinal muscular atrophy Both exons 7 (E7) and 8 (E8) were normal in 98.00% of the screened pregnant women; 1.37% of women had an E7 deletion and E8 heterozygous deletion, 0.08% had an E7 deletion with a normal E8, 0.54% had an E8 heterozygous deletion and a normal E7, and 0.02% had an E8 homozygous deletion and a normal E7

A total of 5,200 pregnant women was screened for SMA from December 1, 2020, to April 21, 2022, using real-time fluorescence quantitative PCR. The age of these women ranged from 16 to 50 years, with an average age of 29.38 ± 5.08 years (Table 1). Of the 5,200 pregnant women tested (Table 2), 75 asymptomatic SMA carriers were identified (Fig. 1), with a carrier rate of 1.44% (95% confidence interval: 1.31–1.65%). Of the 75 identified SMA carriers with a heterozygous deletion of E7 of *SMN1*, 71 patients had both E7 and E8 heterozygous deletions and 4 had an E7 heterozygous deletion with a normal E8. Of the remaining 5,125 women, 28 had a normal E7 with a heterozygous deletion of E8 and 1 had a normal E7 with a homozygous deletion of E8 (Table 3; Fig. 2). Comparison using the χ² test revealed no significant difference in the SMA carrier rates among the different regions of China (χ² = 42, *p* = 0.227).

**Table 1:** Details of the 5,200 pregnant women participating in the screening program

Age group (years)		Number of women	95% confidence interval	
≤ 25		1,235 (23.75%)	23.16–24.34%	
26–34		3,125 (60.10%)	59.42–60.78%	
≥ 35		840 (16.15%)	15.64–16.66%	
Total		5,200 (100.00%)		

**Table 2:** Results of spinal muscular dystrophy carrier screening

Variable	2020.12	2021.01–2021.12	2022.01–2022.04	Total
Screened women (n)	412	3,669	1,119	5,200
Number of carriers	1	50	24	75
Carrier rate	0.24%	1.36%	2.14%	1.44%
95% CI	0.17–0.31%	1.20–1.52%	1.91–2.35%	1.31–1.65%
Partner (n)	0	30	17	47
Recall rate	/	60.00%	70.83%	62.67%
95% CI	/	59.32–60.68%	70.20–71.46%	62.00–63.34%
Carrier couples	/	2	1	3
Prenatal diagnoses (n)	/	2	1	3
Affected cases (n)	/	1	0	1
Pregnancies terminated	/	1	0	1

^CI, confidence interval^

**Table 3:** Results of preliminary screening using real-time fluorescence quantitative polymerase chain reaction

		Exon 8			
		Heterozygous deletion	Homozygous deletion	Normal	Total
Exon 7	Heterozygous deletion	71	0	4	75
	Normal	28	1	5,096	5,125
Total		99	1	5,100	5,200

NGS was used to detect *SMN1* and *SMN2* copy numbers in the screened carriers (Table 4). All 75 carriers had only one copy of *SMN1*. Regarding *SMN2*, of the 75 women, 15 (20.00%) had 1 copy, 31 (41.33%) had 2 copies, and 29 (38.67%) had 3 copies.

**Table 4:** Next-generation sequencing results of spinal muscular dystrophy carriers

	*SMN2* copy number			
*SMN1* copy number	1	2	3	total
1	15 (20.00%)	31 (41.33%)	29 (38.67%)	75

^*SMN*, survival motor neuron^

**Fig. 3:**
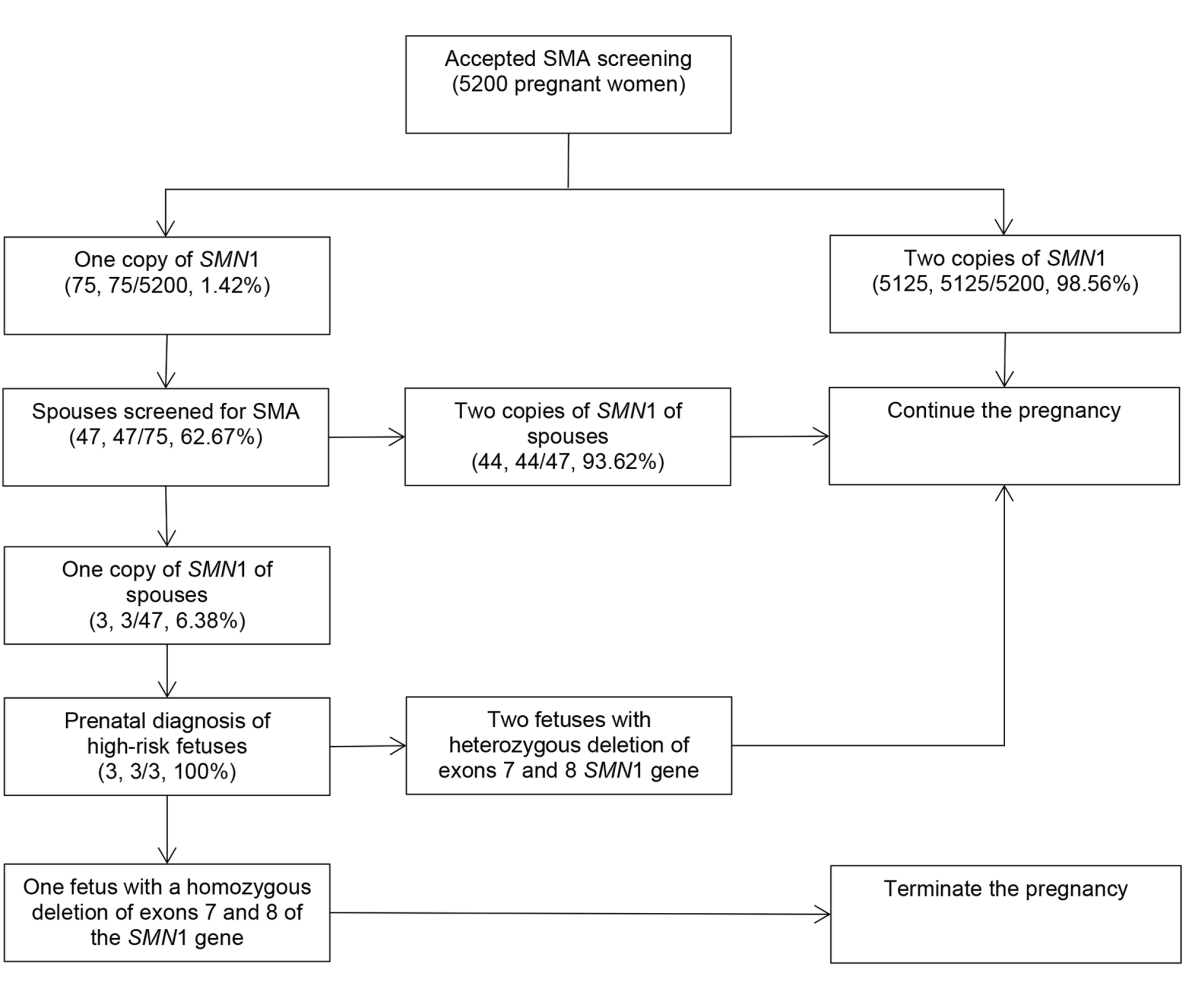
Flowchart for spinal muscular atrophy (SMA) carrier screening and prenatal diagnosis

After screening, the 75 carriers were offered detailed genetic counseling, including information on the etiology, genetic pattern, clinical characteristics, reproductive risk, and treatment of SMA. A total of 47 of these women’s spouses (62.67%) were voluntarily screened for SMA, which revealed that three couples (both spouses) were SMA carriers. Thus, amniocentesis was performed for high-risk fetuses of these three couples. The results revealed that one had a homozygous deletion of E7 and E8 of *SMN1* (Table 5). After offering adequate genetic counseling, the parents of this high-risk fetus decided to terminate the pregnancy. The other two fetuses had a heterozygous deletion of E7 and E8 of *SMN1*, i.e., the SMA carrier genotype; the parents of these fetuses elected to continue their pregnancy (Fig. 3).

**Table 5:** Prenatal diagnosis of fetuses of spinal muscular dystrophy carrier parents

Family	*SMN1* of mother	*SMN1* of father	*SMN1* of fetus	Pregnancy outcomes
1	E7 and E8 heterozygous deletion	E7 and E8 heterozygous deletion	E7 and E8 homozygous deletion	Pregnancy terminated
2	E7 and E8 heterozygous deletion	E7 and E8 heterozygous deletion	E7 and E8 heterozygous deletion	Pregnancy continued
3	E7 and E8 heterozygous deletion	E7 and E8 heterozygous deletion	E7 and E8 heterozygous deletion	Pregnancy continued

^*SMN*, survival motor neuron; E7, exon 7; E8, exon 8^

## Discussion

SMA, a serious autosomal recessive neuromuscular disease, has high carrier rates, and may develop in any ethnic group and present at any age [2]. If both parents are SMA carriers, their offspring have a 25% chance of developing SMA, a 50% chance of being a carrier, and a 25% chance of having a normal genotype.

At present, SMA screening has been conducted in some areas of China [13–18]. In the present study, 5,200 pregnant women were screened for SMA in Zhaoqing city, Guangdong province, which identified 75 SMA carriers. The carrier rate was 1.44%, which is higher than that in Guangxi but lower than that in Taiwan and Yunnan [16, 18] (Table 6). However, the SMA carrier rate among the several domestic regions of China was not significantly different. In most studies on SMA carrier rates, only specific regions were selected for the study. One study that included 34 ethnic groups from 5 provinces of southern China showed that there was no significant difference in the SMA carrier rate among the provinces but that the rate varied among the different ethnic groups [6]. The highest SMA carrier rate was identified in the Tujia ethnicity (4.3%), whereas the lowest rate was identified in the Dai ethnicity (0%), and the rate was 1.4% in the Han ethnicity. These differences may exist because of different sample sizes. In a study on Asians, 1.57% of people had an *SMN1* copy number of 1, which is slightly higher than that detected in the present study, and the carrier rate in other ethnic groups, including Caucasians, Ashkenazi Jews, Hispanics, Asians, Indians, and African Americans, was approximately 0.98–2.02% [4].

**Table 6:** Spinal muscular dystrophy carrier rates in the different regions of China

Area	Survey population (n)	SMA carriers (n)	Carrier frequency of (%)	χ²	*p*	Literature reference
Taiwan	107,611	2,262	1/48 (2.10)	42	0.227	(15)
Shanghai	4,719	90	1/55 (1.9)			(14)
Sichuan	427	9	1/47 (2.11)			(17)
Liuzhou, Guangxi	4,931	61	1/80 (1.2)			(16)
Yunnan	3,049	62	1/49 (2.03)			(18)
Hong Kong	569	9	1/63 (1.6)			(13)
This study	5,200	75	1/69 (1.44)			/

Given the high population carrier rate of SMA and the severity of the disease, screening during pregnancy is critical. Screening can be conducted in three stages. First, pregnant women should be screened for *SMN1* to identify their carrier status. Second, if a pregnant woman is identified as a carrier, her partner should be advised to undergo SMA carrier screening. Finally, if both couples are confirmed as carriers, genetic counseling and prenatal diagnosis should be conducted. Prenatal counseling should include SMA-related information and guidance on genetic etiology, transmission mode, recurrence risk assessment, prenatal diagnosis or preimplantation genetic testing, and carrier screening recommendations for family members.

In the present study, the spouses of the 75 pregnant women who were identified as carriers during screening were also advised to undergo SMA screening. Among them, 47 husbands were tested, with an acceptance rate of 62.67%, and 3 had *SMN1* E7 and E8 heterozygous deletion. After detailed genetic counseling, a prenatal diagnosis of the fetus was recommended. Of the three high-risk fetuses, two had a heterozygous deletion of *SMN1* E7 and E8 and one had a homozygous deletion of *SMN1* E7 and E8, indicating that two fetuses were carriers, and one would have developed SMA after birth. After further genetic counseling, the parents of the latter eventually chose to terminate the pregnancy. There were 28 SMA carriers whose husbands did not participate in screening. Postnatal follow-up was conducted among these women. The results of postnatal follow-up showed that some of the women eventually chose to have their fetuses tested for SMA after birth, whereas the rest of the pregnant women gave birth to children with normal phenotypes. In this study, nearly 30% of the spouses of the pregnant women (carriers) did not undergo SMA screening, indicating that some newborns may have missed the diagnosis. Along with the inclusion of SMA treatment drugs in medical insurance, early detection and early diagnosis via newborn screening and other methods have become important in SMA treatment.

Multiplex ligation-dependent probe amplification (MLPA) is generally acknowledged as the gold standard because of the high degree of precision [19]. However, it requires a long turnaround time and is relatively expensive, making it unsuitable for large-scale SMA carrier screening. In recent years, several studies have used qPCR for carrier screening and the results were verified with MLPA [20, 21]. Among the 75 carriers of pregnant women, only 62.67% of the couples were screened for SMA. The testing costs were also a reason for the low acceptance rate. In a previous study, it was mentioned that in the case of detecting only SMA, three detection technologies, qPCR, NGS, and MLPA, were respectively used to detect SMA, and qPCR had the lowest detection costs [21]. Real-time fluorescence quantitative PCR was used for primary screening in this study as it is simple and inexpensive to perform and suitable for large-scale screening [4, 13, 21]. QPCR can be recommended for detection at other places, so that more pregnant women as well as the general population can undergo SMA screening with lower testing costs.

However, this method can only detect the homozygous deletions of E7 and/or E8 of *SMN1*, not *SMN1* point mutations and “2 + 0"carrier status, which is two SMN1 copies on one chromosome and no copies of the SMN1 gene on the second chromosome. Therefore, in the present study, the possibility of other SMA genotypes could not be excluded when the screening result was negative. During genetic counselling for carrier screening, the following residual risks should be fully explained[9, 22]: quantitative PCR cannot detect approximately 4% of “2 + 0"carrier status in the carrier population [23] as well as approximately 5% of the carriers of the *SMN1* point mutation [24]; about 2% of fetuses with de novo mutations have parents with a normal genotype [25]; and carriers with gonadal chimerism may not be identified via blood-based screening.

In the present study, next-generation sequencing (NGS) was performed to detect *SMN1* and *SMN2* copy numbers in the screening-identified carriers. NGS is suitable for the differential diagnosis of SMA, which includes screening for pathogenic gene variations in patients with non-5q-SMA neuromuscular disease or those with myasthenia as clinical symptoms that need to be excluded. Traditional methods often use different methods to detect different diseases, whereas NGS can accurately screen multiple diseases simultaneously with higher efficiency and lower cost. Recent studies have confirmed that NGS can detect the *SMN1* copy number through improved experimental procedures and bioinformatic analysis [26–30]. In 2017, it was demonstrated that NGS could simultaneously detect the *SMN1* copy number and small *SMN* variants through improved laboratory procedures and bioinformatic analysis [26]. Moreover, in 2020, another study employed NGS for screening *SMN1* and *SMN2* copy numbers with 100% accuracy; the results were validated by multiplex ligation-dependent probe amplification technique-diagnosed known positive cases [27].

The SMA treatment drug nusinersen has been included in medical insurance plans in China; however, despite its inclusion, one dose still costs 33,000 RMB, which is highly expensive for some families. The early symptoms of SMA are not obvious, and affected children are generally tested and diagnosed after birth. Therefore, medical prenatal screening is more economically significant than postnatal treatment. With the advent of drugs, such as nusinersen, postnatal therapy is now available. Many countries already offer newborn screening, and studies have shown that the earlier a child is treated, the better [30–32]. Every effort should be made to diagnose spinal muscular atrophy in the presymptomatic period [31]. Newborn screening will allow more SMA patients to receive timely treatment.

In conclusion, the present study revealed that the SMA carrier rate in Zhaoqing city, Guangdong province, was 1.44% using a large sample size. The screening also helped avoid the birth of one affected fetus via prenatal diagnosis. Reducing the incidence of SMA via pregnancy screening and prenatal diagnosis and advancing the prevention of birth defects can effectively reduce the incidence of birth defects, and reduce the burden on patients’ families. Moreover, research has shown that timely intervention and treatment are critical in improving patient prognosis before the onset of clinical symptoms, which may prevent the development of serious diseases in affected children [32].

## Data Availability

The datasets used and/or analyzed during the current study are available from the corresponding author upon reasonable request.

## References

[1] Lunn MR, Wang CH. Spinal muscular atrophy.Lancet. 2008 Jun21;371(9630):2120–33. 10.1016/S0140-6736(08)60921-6 18572081

[2] Rudnik-Schöneborn S, Forkert R, Hahnen E, Wirth B, Zerres K. Clinical spectrum and diagnostic criteria of infantile spinal muscular atrophy: further delineation on the basis of SMN gene deletion findings. Neuropediatrics. 1996 Feb;27(1):8–15. 10.1055/s-2007-973741 8677029

[3] Sangaré M, Hendrickson B, Sango HA, Chen K, Nofziger J, Amara A, et al. Genetics of low spinal muscular atrophy carrier frequency in sub-saharan Africa. Ann Neurol. 2014 Apr;75(4):525–32. 10.1002/ana.24114 PMC4112719 24515897

[4] Sugarman EA, Nagan N, Zhu H, Akmaev VR, Zhou Z, Rohlfs EM, et al. Pan-ethnic carrier screening and prenatal diagnosis for spinal muscular atrophy: clinical laboratory analysis of > 72 400 specimens. Eur J Hum Genet. 2012 Jan;20(1):27–32. 10.1038/ejhg.2011.134 PMC3234503 21811307

[5] Verhaart IEC, Robertson A, Wilson IJ, Aartsma-Rus A, Cameron S, Jones CC, et al. Prevalence, incidence and carrier frequency of 5q–linked spinal muscular atrophy – a literature review. Orphanet J Rare Dis. 2017 Dec;12(1):124. 10.1186/s13023-017-0671-8 PMC5496354 28676062

[6] Zhao S, Wang W, Wang Y, Han R, Fan C, Ni P, et al. NGS-based spinal muscular atrophy carrier screening of 10,585 diverse couples in China: a pan-ethnic study. Eur J Hum Genet. 2021 Jan;29(1):194–204. 10.1038/s41431-020-00714-8 PMC7852551 32884118

[7] Melki J, Lefebvre S, Burglen L, Burlet P, Clermont O, Millasseau P, et al. De Novo and inherited deletions of the 5q13 region in spinal muscular atrophies. Science. 1994 Jun;3(5164):1474–7. 10.1126/science.7910982 7910982

[8] Wirth B, Herz M, Wetter A, Moskau S, Hahnen E, Rudnik-Schöneborn S et al. Quantitative Analysis of Survival Motor Neuron Copies: Identification of Subtle SMN1 Mutations in Patients with Spinal Muscular Atrophy, Genotype-Phenotype Correlation, and Implications for Genetic Counseling. The American Journal of Human Genetics. 1999 May 1;64(5):1340–56. 10.1086/302369 PMC1377870 10205265

[9] Prior TW. Professional Practice and Guidelines Committee. Carrier screening for spinal muscular atrophy.Genet Med. 2008Nov;10(11):840–2. 10.1097/GIM.0b013e318188d069 PMC3110347 18941424

[10] Committee Opinion No. 691: carrier screening for genetic conditions. Obstet Gynecol. 2017 Mar;129(3):e41–55. 10.1097/AOG.0000000000001952 28225426

[11] The Expert Consensus Group For Preimplantation Genetic Testing For Spinal Muscular Atrophy null, Yan L, Zhu X, Huang J, Qiao J. [Expert consensus on preimplantation genetic testing for spinal muscular atrophy]. Zhonghua Yi Xue Yi Chuan Xue Za Zhi. 2022 Feb;10(2):129–34. 10.3760/cma.j.cn511374-20210118-00048 35076905

[12] Writing Group For Practice Guidelines For Diagnosis And Treatment Of Genetic Diseases Medical Genetics Branch Of Chinese Medical Association null, Pan J, Tan H, Zhou M, Liang D, Wu L. [Clinical practice guidelines for spinal muscular atrophy]. Zhonghua Yi Xue Yi Chuan Xue Za Zhi. 2020 Mar;10(3):263–8. 10.3760/cma.j.issn.1003-9406.2020.03.007 32128742

[13] Chan V, Yip B, Yam I, Au P, Lin CK, Wong V, et al. Carrier incidence for spinal muscular atrophy in southern chinese. J Neurol. 2004 Sep;251(9):1089–93. 10.1007/s00415-004-0487-z 15372251

[14] Gong B, Zhang L, Hou Y, ping, Hu H, Li H, Tan M, yu, et al. [Carrier screening for spinal muscular atrophy in 4719 pregnant women in Shanghai region]. Zhonghua Yi Xue Yi Chuan Xue Za Zhi. 2013 Dec;30(6):670–2. 10.3760/cma.j.issn.1003-9406.2013.06.008 24327144

[15] Su YN, Hung CC, Lin SY, Chen FY, Chern JPS, Tsai C, et al. Carrier screening for spinal muscular atrophy (SMA) in 107,611 pregnant women during the period 2005–2009: a prospective Population-Based Cohort Study. Schrijver I, editor. PLoS ONE. 2011 Feb;25(2):e17067. 10.1371/journal.pone.0017067 PMC3045421 21364876

[16] Tan J, Zhang X, Wang Y, Luo S, Yang F, Liu B et al. [Screening for spinal muscular atrophy mutation carriers among 4931 pregnant women from Liuzhou region of Guangxi]. Zhonghua Yi Xue Yi Chuan Xue Za Zhi. 2018 Aug 10;35(4):467–70. 10.3760/cma.j.issn.1003-9406.2018.04.001 30098235

[17] Zeng G, Zheng H, Cheng J, Chen R, Lin H, Yang J, et al. [Analysis and carrier screening for copy numbers of SMN and NAIP genes in children with spinal muscular atrophy]. Zhonghua Yi Xue Yi Chuan Xue Za Zhi. 2014 Apr;31(2):152–5. 10.3760/cma.j.issn.1003-9406.2014.02.006 24711022

[18] Zhang Y, Wang L, He J, Guo J, Jin C, Tang X, et al. [Result of carrier screening for spinal muscular atrophy among 3049 reproductive-age individuals from Yunnan region]. Zhonghua Yi Xue Yi Chuan Xue Za Zhi. 2020 Apr;10(4):384–8. 10.3760/cma.j.issn.1003-9406.2020.04.005 32219818

[19] Mercuri E, Finkel RS, Muntoni F, Wirth B, Montes J, Main M et al. Diagnosis and management of spinal muscular atrophy: Part 1: Recommendations for diagnosis, rehabilitation, orthopedic and nutritional care. Neuromuscular Disorders. 2018 Feb 1;28(2):103–15. 10.1016/j.nmd.2017.11.005 29290580

[20] Zhang J, Wang Y, Ma D, Sun Y, Li Y, Yang P et al. Carrier Screening and Prenatal Diagnosis for Spinal Muscular Atrophy in 13,069 Chinese Pregnant Women. The Journal of Molecular Diagnostics. 2020 Jun 1;22(6):817–22. 10.1016/j.jmoldx.2020.03.001 32205292

[21] Zhao S, Wang Y, Xin X, Fang Z, Fan L, Peng Z et al. Next generation sequencing is a highly reliable method to analyze exon 7 deletion of survival motor neuron 1 (SMN1) gene.Sci Rep. 2022 Jan7;12(1):223. 10.1038/s41598-021-04325-1 PMC8741787 34997153

[22] Carré A, Empey C (2016). Review of spinal muscular atrophy (SMA) for prenatal and Pediatric genetic counselors. J Genet Couns.

[23] Mailman MD, Hemingway T, Darsey RL, Glasure CE, Huang Y, Chadwick RB, et al. Hybrids monosomal for human chromosome 5 reveal the presence of a spinal muscular atrophy (SMA) carrier with two SMN1 copies on one chromosome. Hum Genet. 2001 Feb;108(2):109–15. 10.1007/s004390000446 11281448

[24] Qu YJ, Bai JL, Cao YY, Wang H, Jin YW, Du J, et al. Mutation spectrum of the survival of Motor Neuron 1 and functional analysis of Variants in chinese spinal muscular atrophy. J Mol Diagn. 2016 Sep;18(5):741–52. 10.1016/j.jmoldx.2016.05.004 27425821

[25] Wirth B, Schmidt T, Hahnen E, Rudnik-Schöneborn S, Krawczak M, Müller-Myhsok B, et al. De novo rearrangements found in 2% of index patients with spinal muscular atrophy: mutational mechanisms, parental origin, mutation rate, and implications for genetic counseling. Am J Hum Genet. 1997 Nov;61(5):1102–11. 10.1086/301608 PMC1716038 9345102

[26] Feng Y, Ge X, Meng L, Scull J, Li J, Tian X, et al. The next generation of population-based spinal muscular atrophy carrier screening: comprehensive pan-ethnic SMN1 copy-number and sequence variant analysis by massively parallel sequencing. Genet Med. 2017 Aug;19(8):936–44. 10.1038/gim.2016.215 28125085

[27] Chen X, Sanchis-Juan A, French CE, Connell AJ, Delon I, Kingsbury Z et al. Spinal muscular atrophy diagnosis and carrier screening from genome sequencing data. Genetics in Medicine. 2020 May 1;22(5):945–53. 10.1038/s41436-020-0754-0 PMC7200598 32066871

[28] Larson JL, Silver AJ, Chan D, Borroto C, Spurrier B, Silver LM. Validation of a high resolution NGS method for detecting spinal muscular atrophy carriers among phase 3 participants in the 1000 Genomes Project. BMC Med Genet. 2015 Oct;29:16:100. 10.1186/s12881-015-0246-2 PMC4625734 26510457

[29] Tan CA, Westbrook MJ, Truty R, Kvitek DJ, Kennemer M, Winder TL et al. Incorporating Spinal Muscular Atrophy Analysis by Next-Generation Sequencing into a Comprehensive Multigene Panel for Neuromuscular Disorders. Genetic Testing and Molecular Biomarkers. 2020 Oct;24(10):616–24. 10.1089/gtmb.2019.0282 32721234

[30] Liu B, Lu Y, Wu B, Yang L, Liu R, Wang H et al. Survival Motor Neuron Gene Copy Number Analysis by Exome Sequencing: Assisting Spinal Muscular Atrophy Diagnosis and Carrier Screening. The Journal of Molecular Diagnostics. 2020 May 1;22(5):619–28. 10.1016/j.jmoldx.2020.01.015 32092542

[31] Jędrzejowska M. Advances in Newborn Screening and Presymptomatic Diagnosis of Spinal Muscular Atrophy.Degener Neurol Neuromuscul Dis. 2020 Dec15;10:39–47. 10.2147/DNND.S246907 PMC7751307 33364872

[32] Mueller-Felber W. Newborn infant screening for spinal muscular atrophy: chances and challenges. Dev Med Child Neurol. 2022 May;64(5):535. 10.1111/dmcn.15152 35156200

